# CD44 Expression Profile Varies According to Maturational Subtypes and Molecular Profiles of Pediatric T-Cell Lymphoblastic Leukemia

**DOI:** 10.3389/fonc.2018.00488

**Published:** 2018-10-31

**Authors:** Luísa Vieira Codeço Marques, Elda Pereira Noronha, Francianne Gomes Andrade, Filipe Vicente dos Santos-Bueno, Marcela B. Mansur, Eugenia Terra-Granado, Maria S. Pombo-de-Oliveira

**Affiliations:** Pediatric Hematology-Oncology Program, Research Center, Instituto Nacional do Câncer, Rio de Janeiro, Brazil

**Keywords:** CD44, CD44/CD44v6, T-cell leukemia, acute myeloid leukemia, *NOTCH1*

## Abstract

CD44 is a glycoprotein expressed in leucocytes and a marker of leukemia-initiating cells, being shown to be important in the pathogenesis of T cell acute lymphoblastic leukemia (T-ALL). In this study, we have (i) identified the aberrant antigenic pattern of CD44 and its isoform CD44v6 in T-ALL; (ii) tested the association with different T-cell subtypes and genomic alterations; (iii) identified the impact of CD44 status in T-ALL outcome. Samples from 184 patients (123 T-ALL and 61 AML; <19 years) were analyzed throughout multiparametric flow cytometry. Mutations in *N/KRAS, NOTCH1, FBXW7* as well as *STIL-TAL1* and *TLX3* rearrangements were detected using standard molecular techniques. CD44 expression was characterized in all T-ALL and AML cases. Compared with AML samples in which the median fluorescence intensity (MFI) was 79.1 (1–1272), T-ALL was relatively low, with MFI 43.2 (1.9–1239); CD44v6 expression was rarely found, MFI 1 (0.3-3.7). T-ALL immature subtypes (mCD3/CD1a^neg^) had a lower CD44 expression, MFI 57.5 (2.7–866.3), whereas mCD3/TCRγδ^pos^ cases had higher expressions, MFI 99.9 (16.4–866.3). *NOTCH1*^mut^ and *STIL-TAL1* were associated with low CD44 expression, whereas *N/KRAS*^mut^ and *FBXW7*^mut^ cases had intermediate expression. In relation to clinical features, CD44 expression was associated with tumor infiltrations (*p* = 0.065). However, no association was found with initial treatment responses and overall survival prediction. Our results indicate that CD44 is aberrantly expressed in T-ALL being influenced by different genomic alterations. Unraveling this intricate mechanism is required to place CD44 as a therapeutic target in T-ALL.

## Introduction

Childhood T-cell acute lymphoblastic leukemia (T-ALL) is an aggressive neoplasm characterized by increased lymphoblast count, mediastinal enlargement, central nervous system infiltration and poor outcome. Despite the great success obtained in ALL treatment along these past three decades, about 30% of T-ALL present high relapse rate with decreased chances of cure (1). Thus, with the better understanding of the T-cell development prognostic indicators, the identification of therapeutic targets in T-ALL are being urged to improve prognostic rates (2).

In this context, CD44 is an adhesion glycoprotein widely expressed in hematopoietic cells, binding to hyaluronic acid and other extracellular matrix components, assisting in the homing and anchorage of HSC to their niche (3, 4). In T-cell development, CD44 has been shown to guide precursors from the bone marrow to the thymus in mouse models (5). In addition to its role as an adhesion and homing molecule, CD44 can also transduce anti-apoptotic and proliferation signaling (6). CD44 is expressed in several malignant hematopoietic disorders, including both T-ALL and acute myeloid leukemia (AML) (7). Although clinically and genetically heterogeneous diseases, T-ALL and AML present overlaps in genetic alterations that lead to pathogenic pathways, such as mutations in genes of the RAS signaling pathway (8), and cellular expression of lineage development markers, such as CD7 expression in AML and CD13 or CD33 in T-ALL (9).

Recently CD44 was also associated with chemotherapy resistance in T-ALL, where its expression was related with the efflux of drugs from the cell *in vitro* (10). The authors also demonstrated that treatment with CD44 blocking antibodies increased the sensitivity of leukemic cells to chemotherapeutics. In addition, CD44 was identified as a direct transcriptional target of NOTCH1 in murine model of T-ALL, where NOTCH1 binding sites were found in the CD44 promoter (11). In the same model, the occupation of leukemic cells in bone marrow, spleen and thymus during disease progression was shown to be dependent on CD44, and the inhibition of CD44 by anti-CD44 antibodies led to the eradication of established T-ALL *in vitro* (11).

Based on this scenario, the aim of this study was to investigate CD44 and its isoform CD44v6 in a cohort of T-ALL cases, in order to determine its aberrant antigenic patterns and its association with different cell maturational subtypes, genetic alterations and clinical characteristics, thus contributing to a better understanding of the role of CD44 in the pathogenesis of T-ALL.

## Materials and methods

### Subjects

Samples from T-ALL and AML patients (< 19 years) as well as 8 umbilical cord blood (UCB) samples were included in this analysis. Bone marrow (BM) aspirates and/or peripheral blood (PB) samples were sent to the Pediatric Hematology-Oncology Research Program, Instituto Nacional de Câncer, Rio de Janeiro, Brazil. Morphology, immunophenotyping by multiparametric flow cytometry (M-FCM), and molecular tests were performed as previously described (12–14). The diagnosis of acute leukemia subtypes followed the World Health Organization (WHO) criteria (15). T-ALL was grouped in immature or mature phenotypes. Immature phenotypes included ETP-ALL, identified according to Coustan-Smith et al. (16) and Inukai et al. (17) criteria and pre-T-ALL, which does not express CD1a and membrane CD3, but expresses other T-cell markers such as CD2, CD5, CD8. Mature phenotype included cortical T-ALL, characterized by cellular expression of CD1a and mature T-ALL characterized by the absence of CD1a and expression of membrane CD3 (15, 18). AML were grouped according to morphological differentiation as minimally differentiated AML (M0, M1), AML with granulocytic differentiation (M2, M3), myelomonoblast AML (M4, M5), and megakaryoblastic AML (M7). Minimally differentiated AML with CD7 positivity were selected, to compare with immature T-ALL cases, as these diseases have many similar pathophysiological features.

The referring physicians provided clinical and demographic data for each case at initial diagnosis, including sex, age, presence of mediastinal mass, central nervous system (CNS) involvement, organomegaly, white blood cell count (WBC) and blast cell count in the PB on day 8 (D8) after 7 days exposure to prednisone. A PB blast count cut-off of 1,000/μL was used to assign patients into two groups; good responders to prednisone (<1,000/μL) and poor responders (>1,000/μL) (19, 20). Patients were not formally enrolled in treatment protocols, but were treated according to either the Brazilian Group for Treatment of Childhood Leukemia (GBTLI-ALL 99 and 2009, *n* = 56), or the Berlin-Frankfurt-Munster ALL (BFM 95 and 2002; *n* = 67) backbone strategies (21, 22).

### Ethics

This study was carried out in accordance with the recommendations of Instituto Nacional de Câncer Research and Ethics Committee with written informed consent from the parents or legal guardians of all subjects. All subjects gave written informed consent in accordance with the Declaration of Helsinki. The protocol was approved by the Instituto Nacional de Câncer Research and Ethics Committee under the registry number CEP/INCA#117/12; CONEP: PB #888.277.

### CD44 and CD44v6 cellular expression

To evaluate CD44 cellular expression, monoclonal antibodies (mAbs) anti-CD44 (G44-26 clone) conjugated to allophycocyanin (APC) fluorochrome were tested on cellular membrane T-ALL and AML blasts and UCB as comparative groups, with follow combinations of mAbs, according to subtype: CD4FITC/CD7PE/CD45PercPCy5.5/CD8PE-Cy7/CD44APC (T-ALL), CD36FITC/CD117PE/CD45PercPCy5.5/HLA-DRPE-Cy7/CD44APC (AML) and CD4FITC/CD34PE/CD45PercPCy5.5/CD8PE-Cy7/CD44APC in control UCB. The monoclonal antibodies were added to 50 μL of BM or 1 × 10^6^ cells, then the staining was performed according to EuroFlow protocol (23). One hundred thousand events were acquired by the FACS Canto-II Flow cytometer (Becton, Dickinson and Company, CA, USA). Data analyses were performed by Infinicyt^TM^ version 1.8 (Cytognos, Salamanca, Spain) Software. CD44 expression was evaluated by median fluorescence intensity (MFI) in blast cells, identified by low/intermediate expression of CD45. CD44v6 FITC (R&D Systems, IgG_1_ Clone #2F10) was also tested in 60 T-ALL samples.

CD44 and CD44v6 MFI normalization was performed in all cases through the ratio between MFI value on leukemic blasts and mononuclear total cells stained with IgG of the same isotype conjugated with APC for CD44 and FITC for CD44v6. The values of CD44 MFI in the median and 75th percentile were used as cutoffs to discriminate between high, intermediary or low CD44 cellular expression.

### Molecular tests

Mutational screening of *NOTCH1* (exons 26, 27 and 34, referent to HD and PEST/TAD domains), *FBXW7* (exons 9 and 10) and *NRAS, KRAS* (codons 12/13) were performed with conditions previously described (12). The presence of *STIL-TAL1* and *TLX3* transcripts were assessed by reverse transcriptase-polymerase chain reaction (RT-PCR) using conditions previously described (24). For all sequencing analyses, the PCR products were purified using the GFX PCR DNA and a Gel Band Purification Kit (GE Healthcare, Amersham, UK) and sequenced using a BigDye Terminator v3.1 Cycle Sequencing Kit (Applied Biosystems, Carlsbad, CA) in a 3500 Genetic Analyzer (Applied Biosystems). The analyses were performed with BioEdit 7.0.9 software, comparing electropherograms with the reference sequences accessed from the National Center for Biotechnology Information (NCBI); *FBXW7* (NM_1013415.1; NG_029466.1), *NOTCH1* (NG_007458.1; NM_017617.3), *KRAS* (NG_7524.1; NM_004985.4) and *NRAS* (NM_002524.4; NG_007572.1).

### Statistical analysis

To compare the distribution of categorical variables, the Chi-square test was used, and Fisher's exact test (2-tailed) was used when the expected count in at least one cell of the table was < 5. Mann-Whitney U test (comparing two Groups) and Kruskal-Wallis test (comparing more than two groups) were used to test for differences in CD44 MFI. The probability of overall survival (OS) was measured from the date of diagnosis to the date of the last follow-up or death from any cause. Relapse-free survival (RFS) was measured from the date of diagnosis to the date of relapse. Patients who did not experience an event or patients lost to follow-up were censored at their date of last known contact. The Kaplan-Meier survival analysis method was used to calculate the OS, and estimated survival values were compared using the log-rank test in order to verify the differences in outcome between CD44 with high, intermediate and low expression. Confidence Intervals (CI) of 95% were used, and *P*-values of < 0.05 were considered statistically significant. Analyses were performed using SPSS 21.0 (SPSS, Chicago, IL, USA, 2004) and PRISM (PRISM, GraphPad, La Jolla, CA, USA) software.

## Results

**Figure 1:**
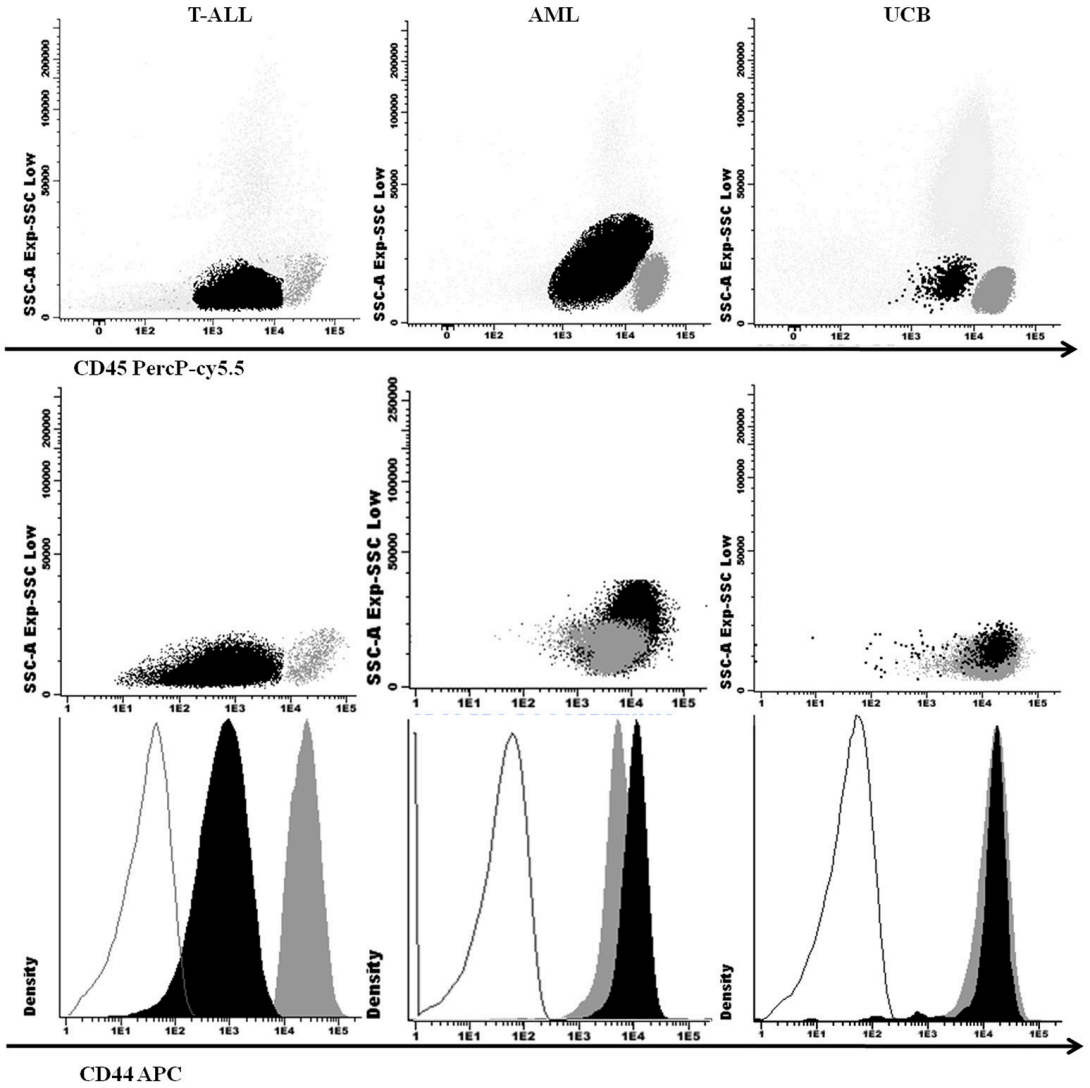
Cellular expression of CD44 in T-ALL, AML, and UCB analyzed by M-FCM. SSC × CD45 (above) and SSC x CD44 (middle) dot plots with leukemic blasts and UCB precursor cells in black and lymphocytes in dark gray. CD44 fluorescence intensity in histograms below, with isotype controls in gray lines, blasts and UCB precursor cells in black and lymphocytes in dark gray. T-ALL, T-cell acute lymphoblastic leukemia; AML, Acute myeloid leukemia; UCB, Umbilical Blood Cord; M-FCM, multiparametric flow cytometry; SSC, side scatter.

One hundred twenty-three pediatric T-ALL and 61 AML cases were included in this study with demographic and clinical features shown in Table 1. Patients with T-ALL and AML had a median age of 8 and 9 years-old, respectively; T-ALL were predominant in males with a ratio of 3.2 males: females (M: F), while in AML patients there was a 1.2 M: F ratio. The higher WBC initial count (≥50 × 10^9^/L) was predominant in T-ALL (*p* < 0.001), as well as organ infiltration presence (*p* < 0.001). Eight UCB samples were analyzed to evaluate the physiological CD44 expression in precursor cells, as shown in Figure 1. The CD45 and CD34 positive precursor cells population was CD44 positive, with a MFI of 194.6 (ranging from 10.7 to 619.1).

**Table 1:** Demographic, clinical features and subtype of patients with T-cell acute lymphoblastic and acute myeloid Leukemia.

	**Total**	**T-ALL**	**AML**	***p***
**AGE (YEARS)**				
< 10	98 (53.3%)	67 (54.5%)	31 (50.8%)	0.75
10–21	86 (46.7%)	56 (45.5%)	30 (49.2%)	
**SEX**				
Males	127 (69%)	94 (76.4%)	33 (54.1%)	0.004
Females	57 (31%)	29 (23.6%)	28 (45.9%)	
**WBC (**×**10**^9^ **/L)**				
<50	55 (30.4%)	25 (20.3%)	30 (49.2%)	< 0.0001
≥50	129 (69.6%)	98 (79.7%)	31 (50.8%)	
**ORGAN INFILTRATIONS**				
Yes	148 (80.1%)	110 (90.2%)	38 (62.3%)	< 0.0001
No	35 (19.9%)	12 (9.8%)	23 (37.7%)	
**SUBTYPE**				
T-ALL				
ETP-ALL		16 (13.2%)	–	
pre-T		26 (21.5%)	–	
Cortical T		42 (34.7%)	–	
Mature T		37 (30.6%)	–	
**AML**				
With minimal differentiation		–	8 (13.1%)	
Without maturation		–	4 (6.6%)	
With maturation		–	7 (11.5%)	
Promyelocytic		–	10 (16.4%)	
Myelomonocytic		–	19 (31.1%)	
Monoblastic		–	8 (13.1%)	
Megakaryoblastic		–	4 (6.6%)	
Total	184	123 (66.8%)	61 (33.2%)	

*T-ALL, T-cell acute lymphoblastic leukemia; ETP, early T-cell precursor; AML, Acute myeloid leukemia; WBC, White blood cell count*.

The immunophenotypic profile is shown in Table 1. Forty-two (34.7%) immature T-ALL (ETP-ALL and pre-T-ALL) and 79 (65.3%) mature T-ALL (cortical T and mature T-ALL) cases were identified; amid AML subtypes, 12 (20.0%) cases were minimally differentiated AML, 17 (28.3%) with granulocytic differentiation, 27 (45.0%) myelomonocyte and 6 (6.7%) were megakaryoblastic. Aberrant phenotype with CD7 antigen was observed in 25.9% of AML samples, mainly M0 subtype.

**Figure 2:**
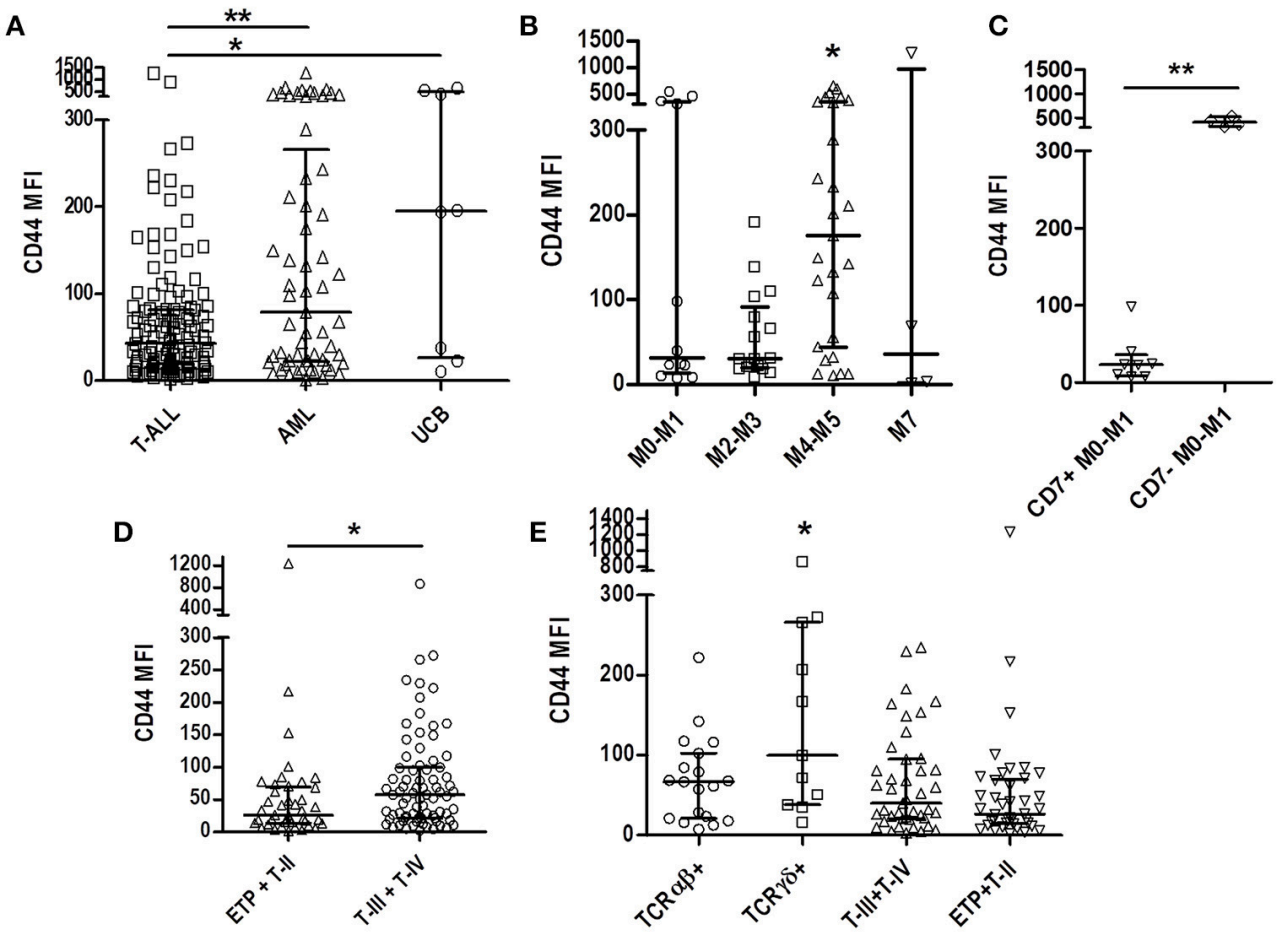
CD44 expression according to T-ALL and AML subtypes and immunophenotypic features. **(A)** Comparison of CD44 expression among T-ALL, AML, and UCB. Scatter dot plot with median and interquartile range of CD44 MFI. **(B)** Comparison of CD44 expression among AML subtypes. Minimally differentiated AML (M0-M1, *n* = 12), AML with granulocytic differentiation (M2-M3, *n* = 17), myelomonocytic (M4-M5, *n* = 27) and megakaryoblastic (M7, *n* = 6). **(C)** Comparison of CD44 expression in minimally differentiated CD7 positive and CD7 negative AML. **(D)** Comparison of CD44 expression between immature T-ALL (ETP and pre-T-ALL, *n* = 42) and mature T-ALL (cortical and mature T-ALL, *n* = 79). **(E)** Comparison of CD44 expression in TCRαβ positive (*n* = 18), TCRδγ positive (*n* = 11) and TCR negative mature (T-III+T-IV, *n* = 43) and immature (ETP+T-II, *n* = 41) cases. T-ALL, T-cell acute lymphoblastic leukemia; AML, Acute myeloid leukemia; UCB, Umbilical cord blood; MFI, Median Fluorescence Intensity; ^*^p < 0.05, ^**^p < 0.01.

We compared the physiological CD44 expression in UCB precursor cells, and CD44 expression in AML blasts, with T-ALL, and observed a lower CD44 expression in T-ALL (*p* = 0.04; Figures 1, 2A).

CD44 was also analyzed in AML samples, as a comparative group, and a MFI of 79.1 (1–1272) was observed, with a higher expression in cases of myelomonocytic AML [M4-M5; MFI 175.5 (10.2-649)] compared to other AML subgroups [AML with minimal differentiation [M0-M1], MFI 31.95 [7.2–545.7], with granulocytic differentiation [M2-M3], MFI 30.0 [7.9-191], and megakaryoblastic AML [M7], MFI 35.5 [1–1272]; *p* = 0.034; Figure 2B. Analyzing CD7 positivity in AML samples with minimally differentiated blast cells, a lower CD44 expression [median 23.0 (7.2–98.3)] was found, compared to CD7 negative AML [median 409.7 (311.1–545.7)] (*p* = 0.004; Figure 2C).

Of the 123 T-ALL cases analyzed for CD44 cellular expression, only one presented < 20% positive blasts, with a percentage median of 95.7% (ranging from 15.6% to 100%) and median MFI of all cases of 43.2 (1.9–1239). To assess CD44 relation with T-ALL blasts' maturational profile, we compared immature T-ALL cases with mature T-ALL cases, and observed a higher CD44 expression in mature cases (MFI 57.5 [2.7–866.3], compared to MFI 26.2 [1.9–1239] ; *p* = 0.013; Figure 2D). Stratifying the mature cases between TCRγδ or TCRαβ positive cases, very high levels of CD44 expression was observed in TCRγδ positive cases MFI 99.9 [16.4–866.3] compared to MFI 66.7 [7.7–222.1] (*p* = 0.014; Figure 2E).

**Figure 3:**
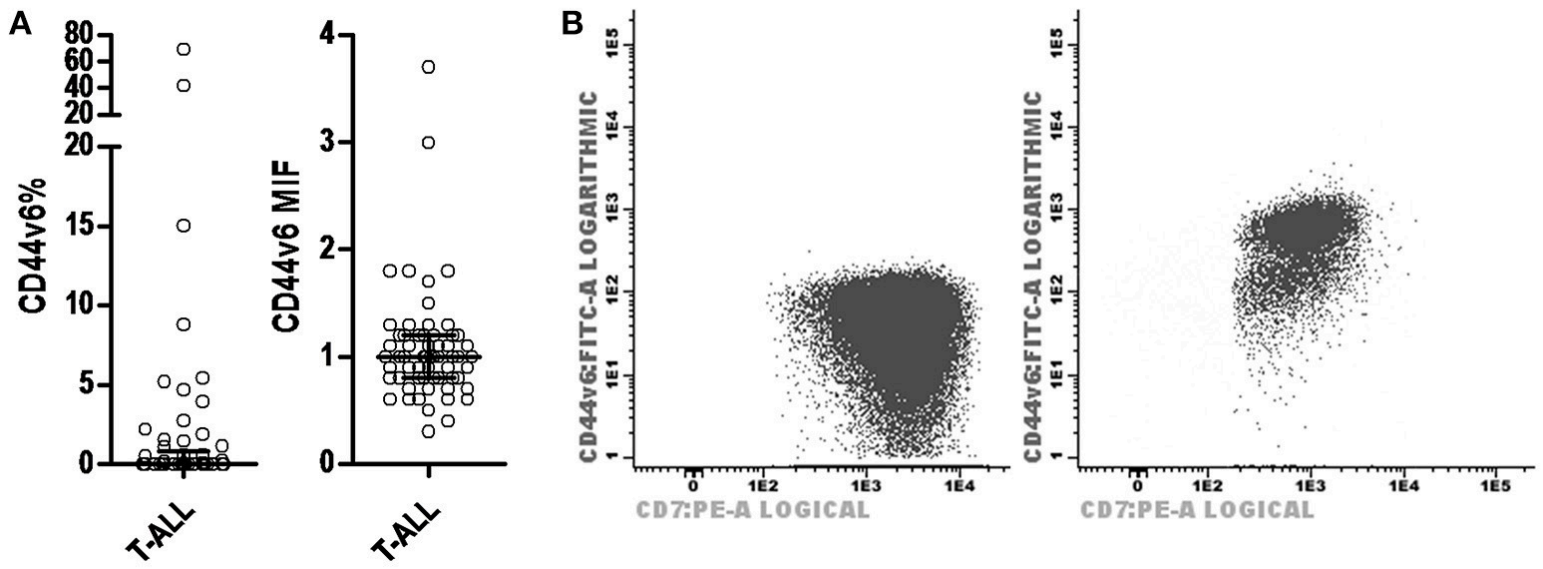
CD44v6 expression profile in T-ALL. **(A)** Scatter dot plots with median and interquartile ranges of CD44v6 percentage of leukemic blasts in T-ALL (on left) and CD44v6 MFI in T-ALL (on right). **(B)** Dot plot CD44v6 × CD7 demonstrating cases of T-ALL with leukemic blasts negative for CD44v6 (on left) and positive for CD44v6 (on right). MFI, median fluorescence intensity; T-ALL, T-cell acute lymphoblastic leukemia.

Regarding CD44v6 expression in T-ALL, of the 61 cases analyzed for this marker the majority did not present expression, with a median of 0% positive cells (0–69.3%) and MFI of 1 (0.3–3.7; Figure 3). The description of cases with CD44v6 expression is shown in Table 2.

**Table 2:** Description of CD44v6 positive T-ALL cases.

**Patient**	**CD44v6 MFI**	**CD44v6%**	**CD44 MFI**	**Age (yrs)/Sex**	**WBC**	Infiltration^*^	**Subtype**	**Molecular alteration^#^**
#1	1.3	0.6	Low	10/M	117,000/μL	Yes	T-IV, TCRγδ	-
#2	1.3	4.0	Interm.	16/M	200,120/μL	No	T-III	-
#3	1.3	2.7	Low	6/M	56,000/μL	Yes	T-III	*NOTCH1*mut; *N/KRAS*mut
#4	1.5	4.7	Low	11/F	250,000/μL	Yes	T-IV, TCRαβ	*NOTCH1*mut; *STIL-TAL1*pos
#5	1.7	5.4	Interm.	0.3/M	399,000/μL	Yes	ETP	-
#6	1.8	8.8	Interm.	17/M	800,000/μL	No	T-IV, TCRαβ	*STIL-TAL1*pos
#7	1.8	15	Interm.	11/M	451,000/μL	Yes	ETP	-
#8	1.8	5.2	Low	13/M	145,600/μL	Yes	T-III	*FBXW7*mut
#9	3.0	41.7	Interm.	7/M	198,000/μL	Yes	T-II	*NOTCH1*mut; *N/KRAS*mut
#10	3.7	69.3	Low	8/M	12,300/μL	Yes	T-III	*STIL-TAL1*pos

* *Mediastinum enlargement, cases #3, #4 and #10; Central nervous system infiltration, case #5. Interm., intermediate; mut, mutation; pos, positive*.

**Figure 4:**
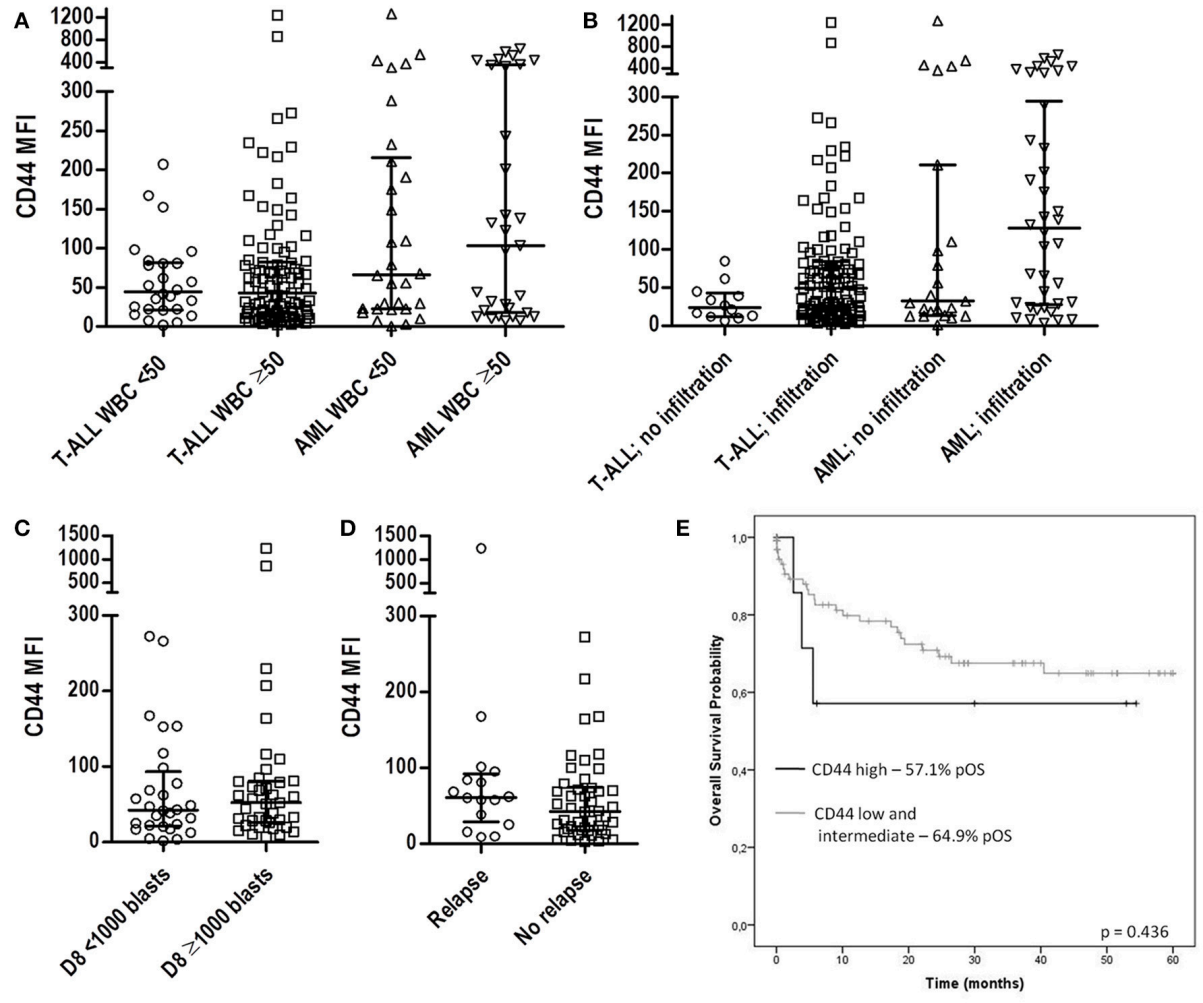
CD44 expression according to patients' clinical characteristics. **(A)** Scatter dot plots with median and interquartile ranges of CD44 MFI in T-ALL and AML according to WBC. Twenty-six T-ALL cases had low WBC (<50 × 10^9^/L) and 97 had high WBC (>50 × 10^9^/L), *p* = 0.877; 30 AML cases had low WBC and 31 high WBC, *p* = 0.702. **(B)** CD44 MFI according to the presence of organ and/or CNS infiltration. In T-ALL, 12 cases did not have the presence of organ infiltration, while 110 had, *p* = 0.0646; in AML, 23 cases did not present organ infiltration, while in 38 it was present, *p* = 0.214. **(C)** CD44 MFI according to prednisone response on D8 in T-ALL. Seventeen cases had good prednisone response and 18 cases poor response, *p* = 0.228. **(D)** CD44 MFI according to occurrance of relapse in T-ALL patients. Sixteen cases presented relapsed T-ALL and 46 no relapse, *p* = 0.278. **(E)** Survival analysis of CD44 expression in T-ALL. Kaplan-Meier estimates for the probability of overall survival for different levels of CD44 expression, *p* = 0.436. All *p*-values calculated by the Mann-Whitney or Log Rank test. T-ALL, T-cell acute lymphoblastic leukemia; AML, Acute myeloid leukemia; MFI, Median Fluorescence Intensity; CNS, Central nervous system; WBC, White blood cell count; pOS, probability of overall survival.

The CD44 expression in T-ALL and AML patients was analyzed according to major clinical signs, such as white blood cell count (WBC), the presence of organ infiltration and prednisone response at day 8 of T-ALL treatment. CD44 MFI was not associated with WBC count in both T-ALL and AML (Figure 4A), while T-ALL cases with organ infiltration presented CD44 MFI 49.4 (1.9–1239) compared to MFI 23.7 [6.7–84.7], (*p* = 0.0646). In AML cases with organ infiltration had CD44 MFI 127.8 (3.1–649), and without infiltration MFI 32.3 (1–1272; *p* = 0.214; Figure 4B). Poor corticoid response was observed in 39 out of 67 (58.2%) and was not significantly associated with CD44 median expression in T-ALL (Figure 4C). T-ALL relapse occurred in 16 cases out of 62 (25.8%) and was also not significantly associated with CD44 median expression (Figure 4D). CD44 expression was not associated with patient's age and sex (Table 3).

**Table 3:** Demographic and clinical characteristics and genomic alterations of T-ALL cases, according to CD44 expression levels.

		**CD44 expression**			
	**Total (%)**	**Low**	**Intermediate**	**High**	***p***
**Age (years)**					
< 10	67 (54.5%)	35 (56.5%)	14 (46.7%)	18 (58.1%)	0.608
10–18	56 (45.5%)	27 (43.5%)	16 (53.3%)	13 (41.9%)	
**Sex**					
Male	94 (76.4%)	44 (71.0%)	25 (83.3%)	25 (80.6%)	0.345
Female	29 (23.6%)	18 (29.0%)	5 (16.7%)	6 (19.4%)	
**WBC**					
<50000/μL	25 (20.3%)	13 (21.0%)	6 (20.0%)	6 (19.4%)	0.982
≥50000/μL	98 (79.7%)	49 (79.0%)	24 (80.0%)	25 (80.6%)	
Organ infiltrations^*^					
**Mediastinum enlargement**					
Yes	45 (36.9%)	23 (37.1%)	9 (30.0%)	13 (43.3%)	0.563
No	77 (63.1%)	39 (62.9%)	21 (70.0%)	17 (56.7%)	
**Hepatomegaly**					
Yes	84 (68.9%)	42 (67.7%)	21 (70.0%)	21 (70.0%)	0.964
No	38 (31.1%)	20 (32.3%)	9 (30.0%)	9 (30.0%)	
**Splenomegaly**					
Yes	91 (74.6%)	43 (69.4%)	23 (76.7%)	25 (83.3%)	0.337
No	31 (25.4%)	19 (30.6%)	7 (23.3%)	5 (16.7%)	
**Lymph node enlargement**					
Yes	84 (68.9%)	42 (67.7%)	21 (70.0%)	21 (70.0%)	0.964
No	38 (31.1%)	20 (32.3%)	9 (30.0%)	9 (30.0%)	
**CNS Infiltration**					
Yes	5 (4.3%)	2 (3.3%)	1 (3.4%)	2 (7.4%)	0.656
No	112 (95.7%)	59 (96.7%)	28 (96.6%)	25 (92.6%)	
***NOTCH1***					
Mut.	49 (47.6%)	29 (56.9%)	11 (44.0%)	9 (33.3%)	0.13
WT	54 (52.4%)	22 (43.1%)	14 (56.0%)	18 (66.7%)	
***FBXW7***					
Mut.	19 (18.4%)	6 (11.8%)	9 (36.0%)	4 (14.8%)	***0.032***
WT	84 (81.6%)	45 (88.2%)	16 (64.0%)	23 (85.2%)	
***STIL-TAL1***					
Positive	33 (27.7%)	21 (35.6%)	4 (13.3%)	8 (26.7%)	0.085
Negative	86 (72.3%)	38 (64.4%)	26 (86.7%)	22 (73.3%)	
***TLX3***					
Positive	6 (5.3%)	4 (7.0%)	1 (3.4%)	1 (3.6%)	0.703
Negative	108 (94.7%)	53 (93.0%)	28 (96.6%)	27 (96.4%)	
***N/KRAS***					
Mut.	13 (10.7%)	5 (8.2%)	7 (23.3%)	1 (3.3%)	***0.029***
WT	108 (89.3%)	56 (91.8%)	23 (76.7%)	29 (96.7%)	
Total	123	62 (50.4%)	30 (24.4%)	31 (25.2%)	

* *One T-ALL case did not have further information of organ infiltrations. T-ALL, T-cell acute lymphoblastic leukemia; WBC, White blood cell count; CNS, Central nervous system; Mut, mutation detected; WT, wild type. Bold and italic values represent p < 0.05*.

**Figure 5:**
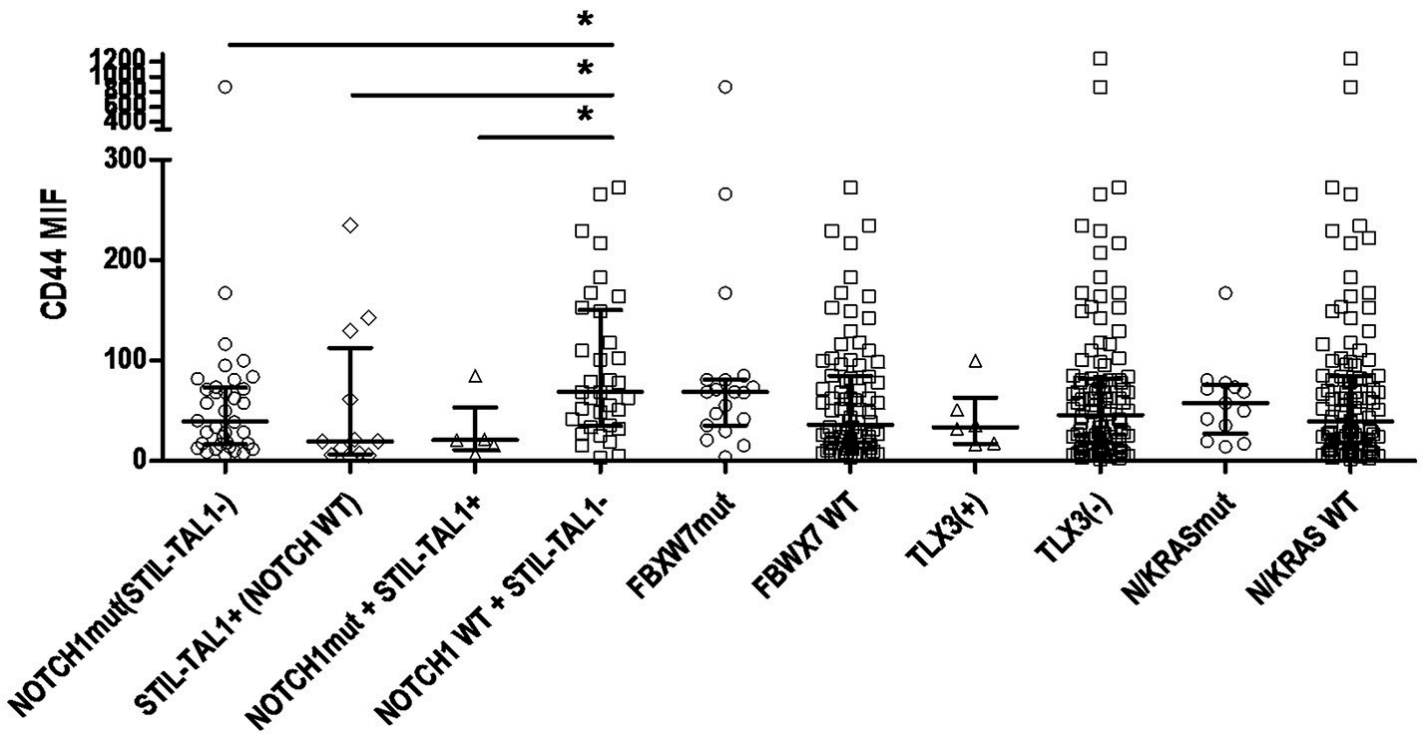
CD44 expression according to status of genomic alterations in T-ALL. Scatter dot plots with median and interquartile ranges of CD44 MFI in T-ALL. Cases with *NOTCH1* mutations and *STIL-TAL1* (−), *n* = 36; *NOTCH1* WT, *STIL-TAL1*(+) cases, *n* = 12; *STIL-TAL1*(+) and *NOTCH1* mutated cases, *n* = 5; *NOTCH1* WT and *STIL-TAL1*(−) cases, *n* = 34; *FBXW7* mutated, *n* = 19; *FBXW7* WT, *n* = 84; *TLX3* positive, *n* = 6; *TLX3* negative, *n* = 108; *N/KRAS* mutated, *n* = 13; WT, *n* = 108. MFI, median fluorescence intensity; mut, mutation; WT, wild type; (+), positive cases; (−), negative cases; ^*^p < 0.05.

T-ALL most common genetic alterations such as *STIL-TAL1* rearrangement, *TLX3* rearrangement, *NOTCH1* and *FBXW7* mutations were assessed, to evaluate CD44 expression levels in different molecular subgroups; 22 cases (21.0%) were *STIL-TAL1* positive and 5.3% *TLX3;* 50 cases (48.5%) had *NOTCH1* mutations, and 19 *FBXW7* mutations. *N/KRAS* mutations and CD44 cellular expression were evaluated in 121 T-ALL and the frequency of *N/KRAS* mutations was 10.7%. In T-ALL, *N/KRAS* and *FBXW7* mutations were associated with an intermediate CD44 expression (*p* = 0.029 and *p* = 0.032, respectively; Table 3). T-ALL cases harboring *NOTCH1* mutations and *STIL-TAL1* positive cases had lower CD44 expression than wild type cases (*NOTCH1* mutated MFI 39.4 [5.8–866.3], *STIL-TAL1* positive MFI 19.3 [3.9–234.7], *NOTCH1* mutated and *STIL-TAL1* positive MFI 20.9 [6.7–84.7], *STIL-TAL1* negative and *NOTCH1* WT MFI 68.75 [3.3–272.3], *p* = 0.017, *p* = 0.031 and *p* = 0.034; Figure 5. *TLX3* had no association with CD44 expression (Table 3).

To assess the value of CD44 in T-ALL as a prognosis marker, we compared overall survival rates between CD44 low, intermediate and high expression groups. Overall, T-ALL cases presented a survival of 56.9%. CD44 levels were not associated with survival in T-ALL (*p* = 0.436; Figure 4E).

## Discussion

Here we explored CD44 cellular expression in a series of childhood T-ALL. Previously, CD44 phenotype in 30 T-ALL cases was positively associated with increased CNS and tumor infiltrations (25). Since then, the mutational landscape of T-ALL was successfully investigated bringing insight into the T-ALL pathogenesis mechanisms, while the CD44 molecule role was unveiled in animal models and in human cell-lines (6, 8, 26, 27). Following the first clinical report on CD44 expression in T-ALL, very few clinical-translational approaches were performed to test the value of CD44 as a marker either in tumor expansion or its relevance in the role of T-ALL outcome. Therefore, the challenge of the present study was to bring more information regarding the relevance of CD44 expression in T-ALL pathogenesis.

Taking into account the normal thymocyte differentiation, CD44 expression is quickly lost in cells in the CD4 CD8 double negative stage and later regained in the double positive stage in T-cells (2). The fact that CD44 positive expression was found in all T-ALL subtypes indicates that CD44 represents an aberrant phenotype in T-ALL and must be considered in the constellation of leukemic markers. In the initial analysis to establish the physiological pattern of CD44 in precursor cells, CD44 expression was screened on CD34 positive UCB precursor cells and also compared to the expression in AML where it was observed that the aberrant expression pattern of CD44 in T-ALL varies from low to intermediate, since normal CD34 positive precursor cells have high expression. AML blasts also had lower expression of CD44 compared to precursor cells of UCB. In the physiological context, the magnitude of CD44 expression is crucial for the differentiation of CD34 positive precursors, where high levels of CD44 lead to differentiation of precursors into monocytes and dendritic cells, and decrease in CD44 expression, differentiation into thymocytes (28). Evaluating CD44 expression in AML maturational subtypes, we found that myelomonocytic AML had the highest CD44 expression among other subtypes, which mirrors the CD44 expression in normal myelomonocytic differentiation, where monocytic precursors show the highest expression of this marker (29). On the other hand, we observed that minimally differentiated AML with CD7 expression and immature T-ALL (ETP and pre-T ALL) had low CD44 expression. Low CD44 expression could then indicate multilineage potential in these diseases.

The highest expression of CD44 in mature subtypes of T-ALL compared to the immature subtypes is similar to the increase in expression that occurs later in differentiation of T cells (2). TCRγδ+ T-ALL had the highest levels of CD44 expression, compared to TCRαβ+ and other T-ALL subgroups. These mature T-ALL with different TCR expressions represent phenotypically and biologically distinct subgroups that differ primarily in relation to specific molecular alterations for each TCR lineage, such as *STIL-TAL1* alteration in αβ-specific lineage, and *TLX3* in γδ-specific lineage (30). These divergent characteristics between TCRαβ or γδ positive T-ALL are also reflected in the different expression of CD44 for each subtype, which makes us consider that CD44 is crucial in the fate of these cells.

Regarding the CD44v6 variant molecule expression, only two cases of T-ALL exhibited more than 20% positive leukemic blasts. CD44v6 was previously associated with the prognosis of patients with AML (29). The almost homogeneous absence of CD44v6 expression in this series of T-ALL cases may be indicative that in the cellular context of T-ALL, the absence of CD44v6 may not play a significant role in the pathogenesis of the disease, but may be interpreted as phenotype aberration. In a study with patients with B-ALL (31), CD44v6 was also rarely expressed, and not associated with survival or relapse of patients, demonstrating that CD44v6 expression does not provide additional information to aid in the designation of patients for specific treatment protocols.

The leukemic transformation of immature thymocytes is the result of a multi-step process involving several different gene alterations that cooperate toward pathogenesis. It is essential to understand the possible interaction between CD44 cell expression and molecular alterations in the T-ALL, resulting in possible therapeutic interventions. With this motivation, we analyzed CD44 expression according to T-ALL genomic alterations, and observed that alterations in *NOTCH1, STIL-TAL1, FBXW7*, and *N/KRAS*, are associated with different phenotypic expression levels of CD44, suggesting that these genes may participate in regulation of CD44.

The low expression of CD44 was associated with *NOTCH1* mutations and the presence of *STIL-TAL1*. CD44 has previously been described as *NOTCH1* transcription target (11), and may explain this association. However, in relation to the TAL1 transcription factor, there are still no studies to date that demonstrate this association with CD44. Thus, these alterations may be interacting with each other and influencing the low expression of CD44. Unraveling this intricate mechanism represents yet another step toward the introduction of new therapeutic approaches in T-ALL.

On the other hand, higher CD44 expression was associated with mutations in *N/KRAS* and *FBXW7*, where the majority of cases with these mutations more frequently had an intermediate CD44 expression. The positive feedback loop between CD44 and the RAS signaling pathway (32) may explain this increase in CD44 expression in *N/KRAS* mutation cases. It is interesting to note that of only two cases with high expression of CD44v6, one of them presented mutation in *N/KRAS*, which corroborates the data described by Cheng et al. (32). As for *FBXW7* mutations, it was previously reported by Babaei-Jadidi et al. (33) an increase in CD44 expression in *FBXW7* deleted intestinal cells in murine models.

In the present study it was observed that the higher expression of CD44 was associated with infiltrations in the patient's organs, which has already been described previously (25), and can be explained by the role of adhesion molecule and homing of CD44. High expressions may also have the future role of predicting hidden infiltrations in patient organs. Although CD44 gene expression was previously described as a predictor of relapse in T-ALL (34), and its associated cell expression and chemoresistance (10), the present study did not observe association between CD44 cellular expression and prednisone response, as well as overall survival of the patient. CD44, then, has not been shown to be useful as a marker of prognosis or response to treatment.

In conclusion, CD44 was highly expressed in pediatric T-ALL whereas the isoform CD44v6 was rarely expressed. CD44 expression was associated with different immunophenotypic profiles and genomic alterations. CD44 was correlated with presence of patients' organ infiltrations, although no association was found with initial response to glucocorticoid, treatment response, and overall survival prediction. These results suggest that CD44 is aberrantly expressed in T-ALL blasts, in different genomic alterational subtypes. Further studies are needed to test the CD44 aspect in homing of leukemic blasts.

## Author contributions

LVCM conducted and analyzed all the experiments and wrote the paper. LVCM, ETG, and EPN performed the immunophenotypic analyses. LVCM, FGA, FVS-B, MBM, and EPN performed the molecular assays. EPN supervised the study, critically analyzed the data, and reviewed the writings. MSPO designed and supervised the study, critically analyzed the data, and reviewed the writings. All authors critically analyzed the data, reviewed the writing, and approved the final draft of the manuscript.

### Conflict of interest statement

The authors declare that the research was conducted in the absence of any commercial or financial relationships that could be construed as a potential conflict of interest.

## References

[1] Van Grotel M Meijerink JP van Wering ER Langerak AW Beverloo HB Buijs-Gladdines JG Prognostic significance of molecular-cytogenetic abnormalities in pediatric T-ALL is not explained by immunophenotypic differences. Leukemia (2008) 22:124–31. 10.1038/sj.leu.2404957 17928886

[2] Canté-Barrett K Mendes RD Li Y Vroegindeweij E Pike-Overzet K Wabeke T. Loss of CD44dim expression from early progenitor cells marks T-cell lineage commitment in the human thymus. Front Immunol. (2017) 8:32. 10.3389/fimmu.2017.00032 28163708 PMC5247458

[3] Ponta H Sherman L Herrlich PA. CD44: from adhesion molecules to signalling regulators. Nat Rev Mol Cell Biol. (2003) 4:33–45. 10.1038/nrm1004 12511867

[4] Zoller M. CD44, hyaluronan, the hematopoietic stem cell, and leukemia-initiating cells. Front Immunol. (2015) 6:235. 10.3389/fimmu.2015.00235 26074915 PMC4443741

[5] Rajasagi M Vitacolonna M Benjak B Marhaba R Zöller M. CD44 promotes progenitor homing into the thymus and T cell maturation. J Leukoc Biol. (2009) 85:251–61. 10.1189/jlb.0608389 18955544

[6] Okamoto I Kawano Y Murakami D Sasayama T Araki N Miki T. Proteolytic release of CD44 intracellular domain and its role in the CD44 signaling pathway. J Cell Biol. (2001) 155:755–62. 10.1083/jcb.200108159 11714729 PMC2150876

[7] Liu J Jiang G. CD44 and hematologic malignancies. Cell Mol Immunol. (2006) 3:359–65. 17092433

[8] Zhang J Ding L Holmfeldt L Wu G Heatley SL Payne-Turner D. The genetic basis of early T-cell precursor acute lymphoblastic leukaemia. Nature (2012) 481:157–63. 10.1038/nature10725 22237106 PMC3267575

[9] Gutierrez A Kentsis A. Acute myeloid/T-lymphoblastic leukaemia (AMTL): a distinct category of acute leukaemias with common pathogenesis in need of improved therapy. Br J Haematol. (2018) 180:919–24. 10.1111/bjh.15129 29441563 PMC5837942

[10] Hoofd C Wang X Lam S Jenkins C Wood B Giambra V. CD44 promotes chemoresistance in T-ALL by increased drug efflux. Exp Hematol. (2016) 44:166–71. e17. 10.1016/j.exphem.2015.12.001 26708679

[11] García-Peydró M Fuentes P Mosquera M García-León MJ Alcain J Rodríguez A. The NOTCH1/CD44 axis drives pathogenesis in a T cell acute lymphoblastic leukemia model. J Clin Invest. (2018) 128:2802–18. 10.1172/JCI92981 29781813 PMC6025994

[12] Mansur MB Hassan R Barbosa TC Splendore A Jotta PY Yunes JA. Impact of complex NOTCH1 mutations on survival in paediatric T-cell leukaemia. BMC Cancer (2012) 12:9. 10.1186/1471-2407-12-9 22225590 PMC3305583

[13] Andrade FG Noronha EP Brisson GD Dos Santos Vicente Bueno F Cezar IS Terra-Granado E. Molecular characterization of pediatric acute myeloid leukemia: results of a multicentric study in Brazil. Arch Med Res. (2016) 47:656–67. 10.1016/j.arcmed.2016.11.015 28476193

[14] Noronha EP Andrade FG Zampier C de Andrade CF Terra-Granado E Pombo-de-Oliveira MS; Brazilian Study Group for Childhood Leukaemia. Immunophenotyping with CD135 and CD117 predicts the FLT3, IL-7R and TLX3 gene mutations in childhood T-cell acute leukemia. Blood Cells Mol Dis. (2016) 57:74–80. 10.1016/j.bcmd.2015.12.003 26852660

[15] Swerlow S Campo E Harris NL Jaffe ES Pileri SA Stein H (2008). WHO Classification of Tumours of Haematopoietic and Lymphoid Tissues. Lyon: International Agency for Research on Cancer 89.

[16] Coustan-Smith E Mullighan CG Onciu M Behm FG Raimondi SC Pei D. Early T-cell precursor leukaemia: a subtype of very high-risk acute lymphoblastic leukaemia. Lancet Oncol. (2009) 10:147–56. 10.1016/S1470-2045(08)70314-0 19147408 PMC2840241

[17] Inukai T Kiyokawa N Campana D Coustan-Smith E Kikuchi A Kobayashi M. Clinical significance of early T-cell precursor acute lymphoblastic leukaemia: results of the Tokyo Children's Cancer Study Group Study L99-15. Br J Haematol. (2012) 156:358–65. 10.1111/j.1365-2141.2011.08955.x 22128890

[18] Bene MC Castoldi G Knapp W Ludwig WD Matutes E Orfao A. Proposals for the immunological classification of acute leukemias. European Group for the Immunological Characterization of Leukemias (EGIL). Leukemia (1995) 9:1783–1786. 7564526

[19] Riehm H Reiter A Schrappe M Berthold F Dopfer R Gerein V. Corticosteroid-dependent reduction of leukocyte count in blood as a prognostic factor in acute lymphoblastic leukemia in childhood (therapy study ALL-BFM 83)]. Klin Padiatr. (1987) 199:151–60. 10.1055/s-2008-1026781 3306129

[20] Reiter A Schrappe M Ludwig WD Hiddemann W Sauter S Henze G. Chemotherapy in 998 unselected childhood acute lymphoblastic leukemia patients. Results and conclusions of the multicenter trial ALL-BFM 86. Blood (1994) 84:3122–3133. 7949185

[21] Scrideli CA Assumpção JG Ganazza MA Araújo M Toledo SR Lee ML. A simplified minimal residual disease polymerase chain reaction method at early treatment points can stratify children with acute lymphoblastic leukemia into good and poor outcome groups. Haematologica (2009) 94:781–9. 10.3324/haematol.2008.003137 19483156 PMC2688569

[22] Stary J Zimmermann M Campbell M Castillo L Dibar E Donska S. Intensive chemotherapy for childhood acute lymphoblastic leukemia: results of the randomized intercontinental trial ALL IC-BFM 2002. J Clin Oncol. (2013) 32:174–184. 10.1200/JCO.2013.48.6522 24344215

[23] Kalina T Flores-Montero J van der Velden VH Martin-Ayuso M Böttcher S Ritgen M. EuroFlow standardization of flow cytometer instrument settings and immunophenotyping protocols. Leukemia (2012) 26:1986–2010. 10.1038/leu,.2012.122 22948490 PMC3437409

[24] Mansur MB Emerenciano M Brewer L Sant'Ana M Mendonça N Thuler LC. SIL-TAL1 fusion gene negative impact in T-cell acute lymphoblastic leukemia outcome. Leuk Lymphoma (2009) 50:1318–25. 10.1080/10428190903040014 19562638

[25] Cavalcanti GB Junior Savino W Pombo-de-Oliveira MS CD44 expression in T-cell lymphoblastic leukemia. Braz J Med Biol Res. (1994) 27:2259–66. 7540475

[26] Demarest RM Ratti F Capobianco AJ. It's T-ALL about Notch. Oncogene (2008) 27:5082–91. 10.1038/onc.2008.222 18758476

[27] Du J Liu Y Meline B Kong G Tan LX Lo JC. Loss of CD44 attenuates aberrant GM-CSF signaling in Kras G12D hematopoietic progenitor/precursor cells and prolongs the survival of diseased animals. Leukemia (2013) 27:754–7. 10.1038/leu.2012.251 22976127 PMC3602969

[28] Williams K Motiani K Giridhar PV Kasper S. CD44 integrates signaling in normal stem cell, cancer stem cell and (pre)metastatic niches. Exp Biol Med. (2013) 238:324–38. 10.1177/1535370213480714 23598979 PMC11037417

[29] Legras S Günthert U Stauder R Curt F Oliferenko S Kluin-Nelemans HC. A strong expression of CD44-6v correlates with shorter survival of patients with acute myeloid leukemia. Blood (1998) 91:3401–13. 9558399

[30] Asnafi V Beldjord K Libura M Villarese P Millien C Ballerini P. Age-related phenotypic and oncogenic differences in T-cell acute lymphoblastic leukemias may reflect thymic atrophy. Blood (2004) 104:4173–80. 10.1182/blood-2003-11-3944 15054041

[31] Khann I Cisterne A Devidas M Shuster J Hunger SP Shaw PJ. Expression of CD44, but not CD44v6, predicts relapse in children with B cell progenitor acute lymphoblastic leukemia lacking adverse or favorable genetics. Leuk Lymphoma (2008) 49:710–18. 10.1080/10428190701861660 18398738

[32] Cheng C Yaffe MB Sharp PA. A positive feedback loop couples Ras activation and CD44 alternative splicing. Genes Dev. (2006) 20:1715–20. 10.1101/gad.1430906 16818603 PMC1522067

[33] Babaei-Jadidi R Li N Saadeddin A Spencer-Dene B Jandke A Muhammad B. FBXW7 influences murine intestinal homeostasis and cancer, targeting Notch, Jun, and DEK for degradation. J Exp Med. (2011) 208:295–312. 10.1084/jem.20100830 21282377 PMC3039859

[34] Yeoh EJ Ross ME Shurtleff SA Williams WK Patel D Mahfouz R. Classification, subtype discovery, and prediction of outcome in pediatric acute lymphoblastic leukemia by gene expression profiling. Cancer Cell (2002) 1:133–43. 10.1016/S1535-6108(02)00032-6 12086872

